# Toward leveraging intrinsic point cloud features in 3D adversarial attacks

**DOI:** 10.1371/journal.pone.0344574

**Published:** 2026-04-21

**Authors:** Hanieh Naderi, Chinthaka Dinesh, Ivan V. Bajić, Shohreh Kasaei

**Affiliations:** 1 College of Interdisciplinary Science and Technologies, University of Tehran, Tehran, Iran; 2 Northeastern University, Vancouver, British Columbia, Canada; 3 School of Engineering Science, Simon Fraser University, Burnaby, British Columbia, Canada; 4 Department of Computer Engineering, Sharif University of Technology, Tehran, Iran; Chunghwa Telecom Co. Ltd., TAIWAN

## Abstract

Adversarial attacks pose serious challenges for deep neural network (DNN)-based analysis of various input signals. In the case of three-dimensional point clouds, methods have been developed to identify points that play a key role in network decision, and these become crucial in generating existing adversarial attacks. For example, a saliency map approach is a popular method for identifying adversarial drop points, whose removal would significantly impact the network decision. This paper seeks to enhance the understanding of three-dimensional adversarial attacks by exploring which point cloud features are most important for predicting adversarial points. Specifically, Fourteen key point cloud features such as edge intensity and distance from the centroid are defined, and random forest regression and multiple linear regression are employed to assess their predictive power for adversarial points. To analyze the potential of intrinsic point cloud features in generating adversarial attacks, we design an attack method. Unlike traditional attack methods that rely on model-specific vulnerabilities, our approach shifts the focus toward the intrinsic characteristics of the point clouds themselves. The proposed attack is tested across four different DNN architectures—PointNet, PointNet++, Dynamic Graph Convolutional Neural Networks (DGCNN), and Point Convolutional Network (PointConv). While its performance is slightly weaker than model-specific attacks, it consistently outperforms random guessing and demonstrates promising generalizability across different models. and demonstrates improved transferability across different architectures. Specifically, the proposed attack achieves on average about a 2% higher success rate in the Drop100 setting and approximately a 4% higher success rate in the Drop200 setting when transferred between models. Beyond adversarial attacks, this study takes a step toward a new perspective in deep learning by shifting the focus from model-specific gradient-based methods to data-driven, feature-based decision-making. This approach has the potential to reduce computational costs by eliminating the need for repeated backpropagation, paving the way for faster and more interpretable deep learning models. These insights can be applied to various domains, including model explainability, feature selection for robust learning, and designing efficient defense mechanisms against adversarial threats.

## 1 Introduction

DNNs have become a go-to approach for many problems in image processing and computer vision [1–5] due to their ability to model complex input-output relationships from a relatively limited set of data. However, studies have also shown that DNNs are vulnerable to adversarial attacks [6,7]. An adversarial attack involves constructing an input to the model (adversarial example) whose purpose is to cause the model to make a wrong decision. Much literature has been devoted to the construction of Two-Dimensional (2D) adversarial examples for image analysis models and the exploration of related defenses [6–10]. Research on adversarial attacks and defenses has gradually expanded to Three-Dimensional (3D) point cloud models as well, especially point cloud classification [11–19].

Point clouds themselves have become an increasingly important research topic [20–23]. Given a deep model for point cloud classification, a number of methods have been proposed to determine critical points that could be used in an adversarial attack [24–26]. For example, Zheng et al. [24] proposed a differentiable method of shifting points to the center of the cloud, known as a *saliency map technique*, which approximates point dropping and assigns contribution scores to input points based on the resulting loss value. Other methods to determine critical points similarly try to estimate the effect of point disturbance on the output.

Once the critical points have been determined, they can be used to create adversarial examples. Several recent studies [11,12,27] use critical points as initial positions and then introduce perturbations to create attacks. Usually, some distance-related criteria, such as Hausdorff [12] or the Chamfer distance [12], are used to constrain perturbations around critical positions. Instead of perturbation, another kind of attack drops the critical points; for example, the well-known Drop100 and Drop200 attacks [24] drop, respectively, 100 and 200 points from the point cloud in order to force the model to make a wrong decision. These are considered to be among the most challenging attacks to defend against [11,28–31].

The methods mentioned above, and others in the literature, require access to the DNN model in order to determine critical points. For example, ”white-box” attacks have access to the model’s internal architecture and parameters, while “black-box” attacks are able to query the model and obtain its output, but without the knowledge of internal details [15]. These approaches are in line with the popular view in the literature [7]: that the existence of adversarial examples is a *flaw of the model*, that they exist because the model is overly parametrized, nonlinear, etc. According to this reasoning, each model has its own flaws, i.e., its own critical points. Another view is that adversarial examples are consequences of the data distribution on which the model is trained [32]. This would suggest that different models trained on the same data may share some adversarial examples, but they have to be determined in the context of the data distribution.

This paper presents a novel perspective, demonstrating for the first time in point cloud literature that adversarially sensitive points can be predicted directly from the intrinsic geometric features of the data—without relying on model architecture or gradient information. We argue that these features capture fundamental shape properties that define object identity — what makes an airplane an airplane, or a chair a chair. Building on this insight, we propose a model-independent attack that uses a feature-based regression framework to identify vulnerable points. The resulting method is lightweight, interpretable, and more transferable across architectures. It unifies concepts from graph signal processing, geometric learning, and adversarial analysis into a coherent framework.

The main contributions of this study are summarized as follows:

- Fourteen geometric features are defined based on graph signal processing to represent the intrinsic structure of 3D point clouds (Sect 3).
- A combination of linear (multiple linear regression) and non-linear (random forest regression) analyses is conducted to identify statistically significant features associated with adversarial drop points.
- A model-independent attack strategy is developed using these features, demonstrating strong generalizability and transferability across different network architectures.
- The robustness of the proposed attack is evaluated under multiple defense mechanisms, showing that geometry-driven point removal remains effective even in the presence of state-of-the-art defenses.

The rest of the paper is organized as follows. The related work is discussed in Sect 2. Our proposed methodology is presented in Sects 3 and 4. The experimental results are reported in Sect 5, followed by conclusions in Sect 6, and future research directions in Sect 7.

## 2 Related work

### 2.1 Deep models for point cloud analysis

PointNet [33] was a pioneering approach for DNN-based point cloud analysis. Learnt features are extracted from individual points in the input point cloud and then aggregated to global features via max-pooling.

As a result of these global features, a shape can be summarized by a sparse set of key points, also called the *critical point set*. The authors of PointNet showed that any set of points between the critical point set and another set called the *upper bound shape* will give the same set of global features, and thus lead to the same network decision. While this proved a certain level of robustness of PointNet to input perturbations, it also pointed to strong reliance on the critical point set, which was subsequently used to design various adversarial attacks.

PointNet has inspired much subsequent work on DNN-based point cloud analysis, of which we review only three approaches subsequently used in our experiments.

One of these is PointNet++ [34], a hierarchical network designed to capture fine geometric structures in a point cloud. Three layers make up PointNet++: the sampling layer, the grouping layer, and the PointNet-based learning layer. These three layers are repeated in PointNet++ to learn local geometric structures.

Another representative work is Dynamic Graph Convolutional Neural Network (DGCNN) [35]. It exploits local geometric structures by creating a local neighborhood graph and using convolution-like operations on the edges connecting neighboring pairs of points.

PointConv [36] is another architecture that extends PointNet by incorporating convolutional layers that work on 3D point clouds. To better handle local features in point cloud data, a multi-layer perceptron (MLP) is used to approximate weight functions, and an inverse density scale is used to re-weight these functions.

### 2.2 Adversarial attacks on point clouds

Deep learning models for 3D point cloud analysis face various security threats, including adversarial attacks [24], backdoor attacks [37], and data poisoning [38]. Some of these, such as backdoor and data poisoning attacks, are introduced during training, whereas this paper specifically focuses on adversarial attacks that occur at test time—that is, attacks in which subtle manipulations of the input data are made after the model has been trained, in order to mislead its predictions. Point clouds are defined by the 3D coordinates of points making up the cloud. Thus, adversarial attacks can be performed by adding, dropping, or shifting points in the input cloud. An adversarial attack can be created by examining all points in the input cloud, or just critical points as potential targets. Liu et al. [11] were inspired by the success of gradient-guided attack methods, such as Fast Gradient Sign Method (FGSM) [6] and Projected Gradient Descent (PGD) [39], on 2D images. They applied a similar methodology to develop adversarial attacks on 3D point clouds. Similarly, the Carlini and Wagner (C&W) [9] optimization for finding adversarial examples has also been transplanted to 3D data. For example, Tsai et al. [40] use the C&W optimization formulation with an additional perturbation-bound regularization to construct adversarial attacks. To generate an attack with a minimum number of points, Kim et al. [41] extend the C&W formulation by adding a term to constrain the number of perturbed points. The adversarial points found in [41] were almost identical to the PointNet critical points.

Xiang et al. [12] demonstrated that PointNet can be fooled by shifting or adding synthetic points or adding clusters and objects to the point cloud. To find such adversarial examples, they applied the C&W strategy to the critical points, rather than all points. Constraining the search space around critical points is sometimes necessary because an exhaustive search through an unconstrained 3D space is infeasible. An attack method that uses the critical-point property for PointNet is proposed by Yang et al. [27]. By recalculating the class-dependent importance for each remaining point, they iteratively remove the most crucial point for the true class. The authors noted that the critical points exist in different models and that a universal point-dropping method should be developed for all models. Wicker et al. [42] proposed randomly and iteratively determining the critical points and then generating adversarial examples by dropping these points.

Arya et al. [43] identify critical points by calculating the largest magnitudes of the loss gradient with respect to the points. After finding those points, the authors propose a minimal set of adversarial points among critical points and perturb them slightly to create adversarial examples. Zheng et al. [24] developed a more flexible method that extends finding critical points to other deep models besides PointNet. They introduced a *saliency score* defined as


$$s_{i}=-r_{i}^{1+\gamma }\frac{\partial ℒ}{\partial r_{i}},$$ (1)

where *r*_*i*_ is the distance of the *i*-th point to the cloud center, γ is a hyperparameter, and $\frac{\partial ℒ}{\partial r_{i}}$ is the gradient of the loss ℒ with respect to the amount of shifting the point towards the center. Adversarial examples are created by shifting the points with high saliency scores towards the center, so that they will not affect the surfaces much.

In addition to the methods developed for creating adversarial attacks on point clouds, some studies [44] have focused on analyzing which local patches contribute most to a model’s decision. Building on such insights, a number of defense mechanisms have also been proposed [15]. For 3D point cloud classification, adversarial training and point removal as a pre-processing step in training have been extensively studied [45]. Some of the methods proposed for point removal to improve robustness against adversarial attacks include simple random sampling (SRS) [27], statistical outlier removal (SOR) [30], Denoiser and UPsampler Network (DUP-Net) [30], high-frequency removal [29], and salient point removal [11].

### 2.3 Explainability methods

Explainability of 3D point cloud deep models is an important emerging area of research. Zhang et al. [24] introduced a class-attentive response map to visualize activated regions in PointNet, while later work [46] focused on interpreting 3D CNNs using statistical methods to evaluate convolution functions. The method in [47] proposed iterative heatmaps to explain point cloud models, and Atkinson et al. [48] introduced a novel classification method that enhances explainability by integrating multiple layers of human-interpretable insights. Other notable approaches include PointMask [49], which used mutual information to mask points, and PointHop [50], which applied local-to-global attributes for classification. More recently, Fan et al. [44] introduced a patch-wise saliency map that highlights important local regions in point clouds by aggregating gradients over neighborhoods.

These methods typically focus on extracting or visualization key point cloud features specific to a particular model, such as PointNet, to improve understanding and explainability. On the other hand, our approach seeks to identify key point cloud features derived from the data’s intrinsic characteristics, making them generalizable across different models.

## 3 Point cloud features

In this section, leveraging recent advances in *graph signal processing* (GSP) [51,52], a set of fourteen features is developed to represent various characteristics of a point cloud. These features will later be analyzed (Sect 4) in terms of their ability to predict adversarial drop points. First, the basic concepts in GSP and the graph construction for a given 3D point cloud are reviewed, leading to the computation of graph-based features.

### 3.1 Preliminaries

#### 3.1.1 Graph definitions.

A undirected weighted graph G=(V,E,W) is defined by a set of *N* nodes V={1,…,N}, edges E={(i,j)}, and a symmetric *adjacency matrix* **W**. $W_{i,j}\in {\mathbb{R}}^{+}$ is the edge weight if (i,j)∈E, and *W*_*i*,*j*_ = 0 otherwise. Diagonal *degree matrix* **D** has diagonal entries $D_{i,i}=\sum_{j}W_{i,j},\forall i$. A *combinatorial graph Laplacian matrix* **L** is defined as L≜D−W  [53]. Further, a *transition matrix* **A** is defined as $\text{A}≜{\text{D}}^{-1}\text{W }$ [54]. By definition $\sum_{j\in {𝒩}_{i}}A_{i,j}=1$. In general, a vector $𝐱=[x_{1}\cdots x_{N}{]}^{⊤}\in {\mathbb{R}}^{V}$ can be interpreted as a graph signal, where *x*_*i*_ is a scalar value assigned to node i∈V. Further, for a given graph signal **x**, a weighted average signal value at node *i* around its neighbors can be computed as


$${\bar{x}}_{i}=(\text{Ax}{)}_{i}=\sum_{j\in N_{i}}A_{i,j}x_{j},$$ (2)

where $N_{i}$ is the 1-hop neighborhoods of node *i*. Moreover, the second difference of the graph signal **x** at node *i* is given as


$${\tilde{x}}_{i}=(\text{Lx}{)}_{i}=L_{i,i}x_{i}+\sum_{j\in N_{i}}L_{i,j}x_{j}.$$ (3)

#### 3.1.2 Graph construction for a 3D point cloud.

To enable graph-based feature-extraction of *n* 3D points, a neighborhood graph is first constructed. In particular, an undirected positive graph 𝒢=(𝒱,ℰ,W) is created, consisting of a node set 𝒱 of size *n* (where each node represents a 3D point) and an edge set ℰ defined by (*i*,*j*,*W*_*i*,*j*_), where i≠j, i,j∈𝒱, and $W_{i,j}\in {\mathbb{R}}^{+}$.

Each 3D point (graph node) is connected to its *k* nearest neighbors *j* in Euclidean distance, so that each point can be filtered with its *k* neighboring points under a graph-structured data kernel [53,55].

In the graph-based point cloud processing literature [56–59], using pairwise Euclidean distance $\|{\text{p}}_{i}-{\text{p}}_{j}{\|}_{2}^{2}$ to compute edge weight *W*_*i*,*j*_ between nodes *i* and *j* is popular, *i.e.*,


$$W_{i,j}=\exp\left\{-\frac{{\left\|{𝐩}_{i}-{𝐩}_{j}\right\|}_{2}^{2}}{\sigma^{2}}\right\},$$ (4)

where ${𝐩}_{i}\in {\mathbb{R}}^{3}$ is the 3D coordinate of point *i* and σ is a parameter. In numerous graph-based point cloud processing works [56–58,60–64], σ was manually chosen so that edge weight *W*_*i*,*j*_ is large (close to 1) if 3D points *i*, *j* are physically close, and small (close to 0) otherwise.

### 3.2 Graph-based feature extraction

Based on these notions, three sets of features are computed for each 3D point in a given point cloud as follows.

#### 3.2.1 3D point coordinates-based features.

A point cloud $\text{P}\in {\mathbb{R}}^{n\times 3}$ is a set of *n* points in a 3D space, **P** = [**p**_*i*_], *i* = 1,2,..., *n*, where each point, ${\text{p}}_{i}=(p_{x,i},p_{y,i},p_{z,i})$, is represented by its x-y-z coordinates. The point cloud can also be represented as $\text{P}=[{\text{p}}_{x};{\text{p}}_{y};{\text{p}}_{z}]$, where **p**_*x*_, **p**_*y*_, and **p**_*z*_ are the *n*-dimensional column-vectors representing *x*-, *y*-, and *z*-coordinates. Here, we consider **p**_*x*_, **p**_*y*_, and **p**_*z*_ as three graph signals on graph G constructed from a given point cloud as in Sect 3.1.2. Therefore, using (2), one can easily compute the weighted average 3D coordinate at node *i* as follows:


$${\bar{\text{p}}}_{i}=\left({\left(\text{AP}\right)}^{⊤}\right)(:,i)=\sum_{j\in {𝒩}_{i}}A_{i,j}{\text{p}}_{j},$$ (5)

where $\left({\left(\text{AP}\right)}^{⊤}\right)(:,i)$ is the *i*-th column of ${\left(\text{AP}\right)}^{⊤}$. Further, according to (3), the second difference of 3D coordinates **P** at node *i* can be written as follows:


$${\tilde{\text{p}}}_{i}=\left({\left(\text{LP}\right)}^{⊤}\right)(:,i)=L_{i,i}{\text{p}}_{i}+\sum_{j\in {𝒩}_{i}}L_{i,j}{\text{p}}_{j}.$$ (6)

Now, the *x*-, *y*-, and *z*-coordinates ${\bar{\text{p}}}_{i}$ and ${\tilde{\text{p}}}_{i}$ are considered as one set of graph-based features at point *i*.

#### 3.2.2 Local variation-based features.

Similar to [54], local variation at point *i* can be quantified as:


$$v_{i}={\left\|{\text{p}}_{i}-{\bar{\text{p}}}_{i}\right\|}_{2},$$ (7)

where ${\bar{\text{p}}}_{i}$ is in (5). Here, one can easily see that *v*_*i*_ is the Euclidean distance between point **p**_*i*_ and the weighted average of its neighbors. Therefore, when the value of *v*_*i*_ is high, the point *p*_*i*_ cannot be well approximated from those of its neighboring points, and hence the point **p**_*i*_ is thus likely to be a point of an edge, corner, or valley.

Further, we consider $\text{v}=[v_{1}\cdots v_{n}{]}^{⊤}$ as a graph signal of the graph G constructed from the given point cloud as discussed in Sect 3.1.2. Then, according to (2), weighted average signal value at node *i* is given as:


$${\bar{v}}_{i}=\sum_{j\in {𝒩}_{i}}A_{i,j}v_{j}.$$ (8)

Using (3), second difference of signal **v** at node *i* is given as:


$${\tilde{v}}_{i}=L_{i,i}v_{i}+\sum_{j\in {𝒩}_{i}}L_{i,j}v_{j}.$$ (9)

Now, we consider *v*_*i*_, ${\bar{v}}_{i}$ and ${\tilde{v}}_{i}$ are the second set of graph-based features at point *i*.

#### 3.2.3 Low-pass filter-based features.

For graph signals **p**_*x*_, **p**_*y*_, **p**_*z*_ with respect to the graph G, the corresponding *low-pass filter* (LPF) signals ${\text{q}}_{x}^{*}$, ${\text{q}}_{y}^{*}$, ${\text{q}}_{z}^{*}$ can be obtained by minimizing the following optimization problem (See [65] for details):


$${\text{q}}^{*}=\arg\min_{\text{q}}{\left\|\text{p}-\text{q}\right\|}_{2}^{2}+\gamma \left({\text{q}}_{x}^{⊤}\text{L}{\text{q}}_{x}+{\text{q}}_{y}^{⊤}\text{L}{\text{ q}}_{y}+{\text{q}}_{z}^{⊤}\text{L}{\text{q}}_{z}\right),$$ (10)

where ${\text{q}}^{*}=[{\left({\text{q}}_{x}^{*}\right)}^{⊤}\;{\left({\text{q}}_{y}^{*}\right)}^{⊤}\;{\left({\text{q}}_{z}^{*}\right)}^{⊤}{]}^{⊤}$, $\text{q}=[{\text{q}}_{x}^{⊤}\;{\text{q}}_{y}^{⊤}\;{\text{q}}_{z}^{⊤}{]}^{⊤}$, $\text{p}=[{\text{p}}_{x}^{⊤}\;{\text{p}}_{y}^{⊤}\;{\text{p}}_{z}^{⊤}{]}^{⊤}$, and γ>0 is a regularization parameter. Since (10) is a *quadratic programming* (QP) problem, its solution can be obtained by solving the following system of linear equations:


$$\left(\text{I}+\gamma \bar{\text{L}}\right){\text{q}}^{*}=\text{p},$$ (11)

where $\bar{\text{L}}=\text{diag}\{\text{L},\text{L},\text{L}\}$. Since **L** is a *positive semi-definite* (PSD) matrix by definition [66], one can easily see that $\bar{\text{L}}$ is also a PSD matrix. Hence $\left(\text{I}+\gamma \bar{\text{L}}\right)$ is a *positive definite* (PD) matrix for γ>0 and thus invertible.

Moreover, we define another graph signal $\text{h}=[h_{1}\cdots h_{n}]$, where $h_{i}={\left\|{\text{p}}_{i}-{\text{q}}_{i}^{*}\right\|}_{2}$. Here, ${\text{q}}_{i}^{*}\in {\mathbb{R}}^{3}$ is *x*, *y*, *z* coordinates of the LPF coordinates at point *i* obtained from (11). Further, similar to (8) and (9), we can compute the weighted average signal value at node *i* and the second difference of **h** at node *i* (denoted as ${\bar{h}}_{i}$ and ${\tilde{h}}_{i}$, respectively.) Then, we consider *h*_*i*_, ${\bar{h}}_{i}$ and ${\tilde{h}}_{i}$ are the third set of graph-based features at point *i*.

In addition to the aforementioned graph-based features, the following two features are computed for each point *i*:

1. Euclidean distance between point *i* and the centroid of the corresponding point cloud.
2. The number of points inside a ball of radius *r* and center **p**_*i*_. In this paper, we manually choose *r* = 0.1.

Altogether, fourteen features (denoted as $\left\{f_{j}^{i}\;|\;j=1,...,14\right\}$) have been created for any given point *i*, as summarized in Table 1. Table 1. These are: the edge intensity value ($f_{1}^{i}$), weighted average of 3D coordinates around neighboring points ($f_{2}^{i}$, $f_{3}^{i}$, $f_{4}^{i}$), second difference of 3D coordinates ($f_{5}^{i}$, $f_{6}^{i}$, $f_{7}^{i}$), the weighted average of edge intensity values around neighboring points ($f_{8}^{i}$), the second difference of edge intensity values ($f_{9}^{i}$), the distance from the centroid ($f_{10}^{i}$), the number of points inside a ball of radius *r* = 0.1 ($f_{11}^{i}$), the distance between actual 3D points and the low-pass-filtered (LPF) 3D points ($f_{12}^{i}$), the weighted average of LPF distance around neighbors ($f_{13}^{i}$) and the second difference of LPF distance ($f_{14}^{i}$).

**Table 1:** Fourteen features created for the *i*-th point in a given point cloud.

Feature symbol	Explanation
$f_{1}^{i}$	*v*_*i*_. See (7) and Sect 3.2.2.
$f_{2}^{i}$	*x* coordinate of ${\bar{𝐩}}_{i}$. See (5) and Sect 3.2.1.
$f_{3}^{i}$	*y* coordinate of ${\bar{𝐩}}_{i}$. See (5) and Sect 3.2.1.
$f_{4}^{i}$	*z* coordinate of ${\bar{𝐩}}_{i}$. See (5) and Sect 3.2.1.
$f_{5}^{i}$	*x* coordinate of ${\tilde{𝐩}}_{i}$. See (6) and Sect 3.2.1.
$f_{6}^{i}$	*y* coordinate of ${\tilde{𝐩}}_{i}$. See (6) and Sect 3.2.1.
$f_{7}^{i}$	*z* coordinate of ${\tilde{𝐩}}_{i}$. See (6) and Sect 3.2.1.
$f_{8}^{i}$	${\bar{v}}_{i}$. See (8) and Sect 3.2.2.
$f_{9}^{i}$	${\tilde{v}}_{i}$. See (9) and Sect 3.2.2.
$f_{10}^{i}$	Euclidean distance between point *i* and the centroid of the point cloud.
$f_{11}^{i}$	The number of points inside a ball of radius *r* and center **p**_*i*_.
$f_{12}^{i}$	*h*_*i*_. See Sect 3.2.3.
$f_{13}^{i}$	${\bar{h}}_{i}$. See Sect 3.2.3.
$f_{14}^{i}$	${\tilde{h}}_{i}$. See Sect 3.2.3.

## 4 Feature analysis and new attack

In this section, a methodology is presented to examine how well the features introduced in Sect 3 are able to predict adversarial drop points. First, each point is assigned an *adversarial score* that reflects how much it contributes to the corresponding model’s prediction. Then, both linear and non-linear regression models are employed to examine how predictive the proposed features are of the adversarial score.

### 4.1 Adversarial score

Let $ϝ:{\mathbb{R}}^{n\times 3}\to \{1,2,...,C\}$ be a trained *C*-class classifier, which maps an input point cloud **P** to a class $c_{t}\in \{1,2,...,C\}$, such that $ϝ(\text{P})=c_{t}$. An adversarial attack aims to deceive the classifier by changing the point cloud **P** to **P**^*adv*^ so that $ϝ({\text{P}}^{adv})\neq c_{t}$, while usually also requiring ${\text{P}}^{adv}\approx \text{P }$.

Saliency score (1) is an indicator of the sensitivity of the classifier to a perturbation of an input point. It has been shown [24] to be effective in determining adversarial points, in the sense that perturbing or removing points with a high saliency score can create adversarial examples. One issue with the saliency score is that its dynamic range can be variable. Hence, the adversarial score is defined by normalizing the saliency score to the range [0, 1]. Specifically, for the *i*-th point **p**_*i*_, the adversarial score is defined as


$$z({\text{p}}_{i})=\frac{s_{i}-\min\{s_{1},...,s_{n}\}}{\max\{s_{1},...,s_{n}\}-\min\{s_{1},...,s_{n}\}},$$ (12)

where *s*_*i*_ is given in (1). In the next section, we examine how well our fourteen features are able to predict *z*(**p**_*i*_).

### 4.2 Multiple linear regression

Multiple linear regression analysis [67,68] is employed to examine how predictive are the features defined in Sect 3 of the adversarial score *z*(**p**_*i*_) in (12). Note that this idea may seem strange to start with: *z*(**p**_*i*_) depends on the classifier model, via the loss ℒ in the saliency score *s*_*i*_ in (1), while the features in Sect 3 do not! Yet, as the results will show, some of our features are fairly predictive of *z*(**p**_*i*_).

**Fig 1:**
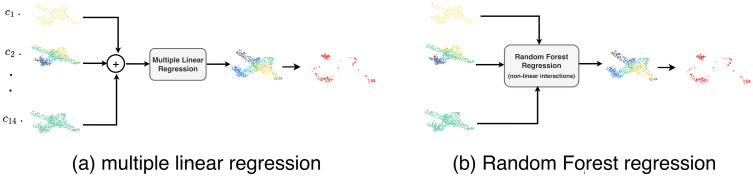
An overview of feature-based adversarial point analysis using (a) multiple linear regression and (b) random forest regression. Fourteen geometric features are computed for each point in the point cloud. In the figure, feature values are represented with different colors, with black corresponding to high values and light yellow corresponding to low values. In (a), at each point, features are linearly combined using the coefficients estimated on the training data, whose significance is determined using statistical testing. In (b), a random forest regressor is employed to model non-linear interactions among the same features and estimate point-wise adversarial relevance. In both cases, the points with the highest predicted scores are selected and compared against the true adversarial drop points.

For a given point **p**_*i*_, its adversarial score *z*(**p**_*i*_) and its features $\left\{f_{j}^{i}\;|\;j=1,...,14\right\}$, we set up the following multiple linear regression model:


$$z({\text{p}}_{i})\approx \sum_{j=1}^{14}c_{j}\cdot f_{j}^{i},$$ (13)

where *c*_*j*_ are the regression coefficients. A conceptual illustration of this formulation is shown in Fig 1(a). To determine whether a particular feature is predictive of *z*(**p**_*i*_), we perform a hypothesis test for each coefficient *c*_*j*_ in the form of a two-tailed t-test [67]:


$$H_{0}:c_{j}=0,\;H_{1}:c_{j}\neq 0.\;$$ (14)

Here, the null hypothesis *H*_0_ is *c*_*j*_ = 0. If the null hypothesis cannot be rejected for a particular coefficient *c*_*j*_, it means that, given the variation, the coefficient is not significantly different from zero. The interpretation would be that the corresponding feature $f_{j}^{i}$ does not contribute significantly to the prediction of the adversarial score *z*(**p**_*i*_). On the other hand, for coefficients *c*_*j*_ where the null hypothesis can be rejected, we can conclude that the corresponding feature $f_{j}^{i}$ is statistically significantly predictive of *z*(**p**_*i*_).

### 4.3 Random forest regression

While multiple linear regression provides a transparent and interpretable framework for analyzing the relationship between geometric features and adversarial scores, it is inherently limited to linear dependencies. To further investigate whether non-linear interactions between features play a role in predicting adversarial points, we additionally employ random forest regression as a complementary analysis. Random forest regression is an ensemble-based non-linear learning method that combines multiple decision trees to model complex relationships between input variables and a target output (see Fig 1(b)). Unlike linear regression, random forests do not assume a predefined functional form between features and the response variable, and are capable of capturing higher-order interactions and non-linear effects among features. Given the same adversarial score *z*(**p**_*i*_) defined in (12) and the set of fourteen geometric features $\left\{f_{j}^{i}|j=1,\ldots ,14\right\}$, the random forest model learns a non-linear mapping


$$z({\text{p}}_{i})\approx ℱ(f_{1}^{i},f_{2}^{i},\ldots ,f_{14}^{i}),$$ (15)

where ℱ(·) represents an ensemble of decision trees trained on randomly sampled subsets of data and features. In addition to prediction accuracy, an important advantage of random forest regression is its ability to estimate feature importance. Feature importance scores are computed based on the average reduction in prediction error achieved by splits involving each feature across all trees in the ensemble. These scores provide a quantitative measure of how strongly each feature contributes to predicting adversarial scores, without relying on linear assumptions. Random forest regression is used as a complementary non-linear validation to confirm whether features identified by the linear model remain influential under non-linear dependencies.

### 4.4 New drop attack

Based on the combined analysis in Sects 4.2 and 4.3, we focus on a single dominant feature from this set. For each point **p**_*i*_ in a given point cloud **P**, we compute the predicted adversarial score as


$$𝔷({\text{p}}_{i})=f_{10}({\text{p}}_{i}),$$ (16)

where $f_{10}({\text{p}}_{i})$ denotes the distance of point **p**_*i*_ from the centroid of the point cloud. Note that *z*(**p**_*i*_) is the true adversarial score obtained from (1) and (12), which requires access to the target DNN model, while $𝔷({\text{p}}_{i})$ is the predicted adversarial score computed from the point cloud itself.

A drop-*N* attack is defined by removing a subset of points with the highest predicted vulnerability, based solely on intrinsic geometric feature *f*_10_ and without relying on model-specific gradients.

## 5 Experiments

### 5.1 Experimental setting

The aligned benchmark ModelNet40 dataset, containing 40 object classes, was used. The dataset employed in this study consists of 9,843 training and 2,468 test point clouds. Each point cloud contains 1,024 points. We employed the standard augmented version of the dataset, where 1024 points are randomly sampled from each object’s surface and normalized to fit within a unit sphere. This preprocessing ensures consistent scale and point density across all objects. For start, three models – PointNet, PointNet++, and DGCNN, with implementation from [28] – were used as deep classifiers on the ModelNet40 dataset. Subsequently, another model, PointConv [36], was used to test attack transferability.

All experiments were conducted using PyTorch on a machine with a NVIDIA Tesla P100-PCIe card with 16 GB of memory.

### 5.2 Feature-based adversarial point analysis

**Fig 2:**
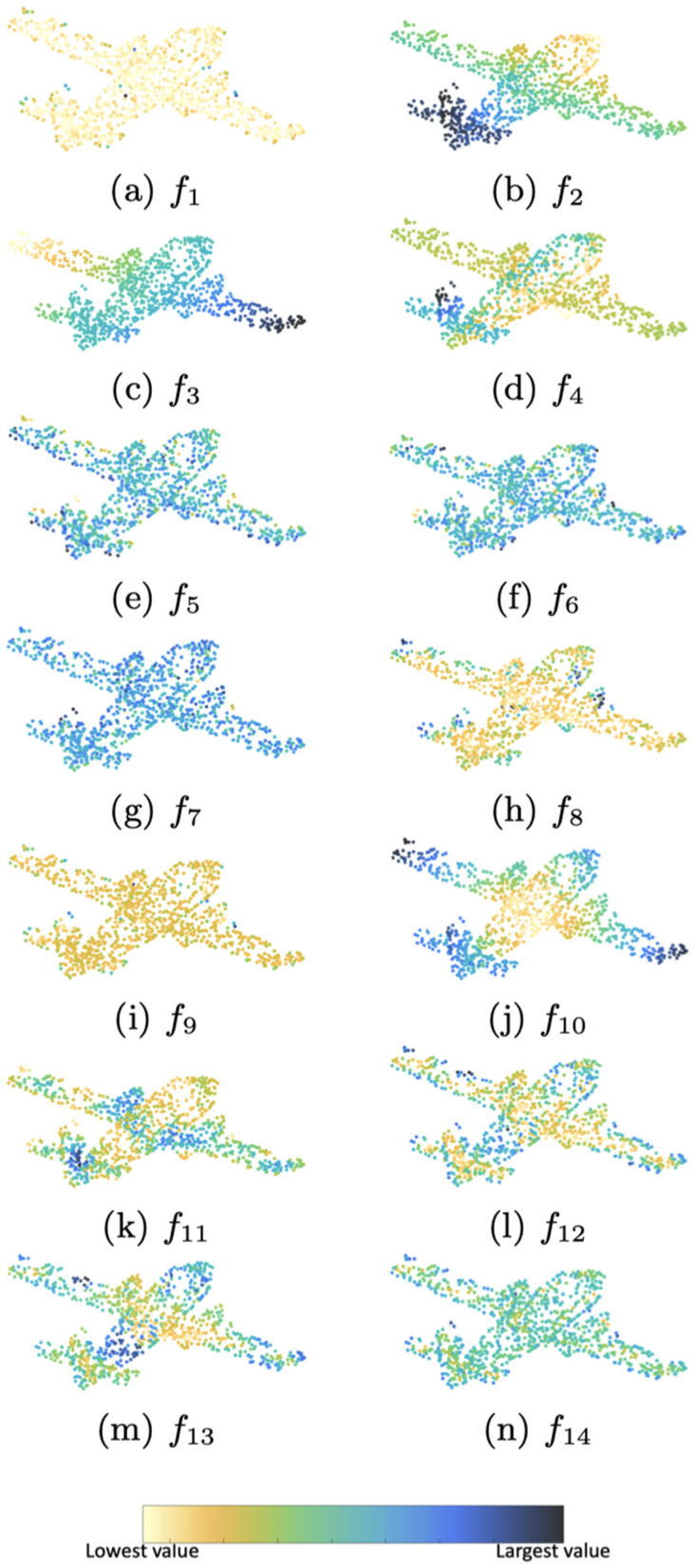
Visualization of fourteen features. Points are colorized by the feature value at each point, according to the shown color map.

Fig 2 shows a visualization of the fourteen features defined in Sect 3 on the airplane object. Each point’s color reflects the value of the corresponding feature at that point, with dark blue indicating high values and light yellow indicating low values.

The approach from [24] was used to compute saliency scores (1) iteratively. Specifically, saliency scores were first computed for the initial point cloud; then, the top 10 points with the highest score were removed. This process was repeated on the remaining points until a total of *N* points were identified. Since high saliency scores are more reliable, these top-*N* points were then used to compute their adversarial scores *z*(**p**_*i*_) as in (12).

Subsequently, these *N* points, where N∈{50,100,150,200}, are used as the basis for feature-based adversarial point analysis using both linear (multiple linear regression) and non-linear (random forest regression) models.

**Fig 3:**
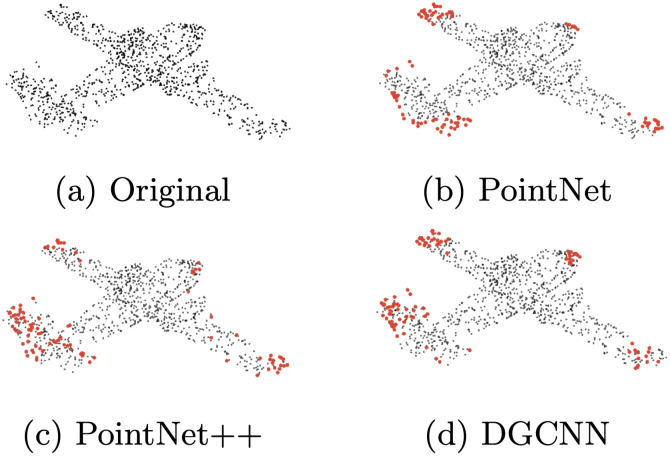
Illustration of adversarial points on the airplane object (a); sub-figures (b), (c), and (d) show the 100 points with the highest adversarial score obtained using the corresponding network.

Fig 3 shows a visualization of the 100 points with the highest adversarial score, computed from saliency scores of three models: PointNet, PointNet++, and DGCNN.

An interesting observation is that, while each network is different, adversarial points tend to cluster in specific regions like the tail, wings, and tips. Comparing Figs 2 and 3, reveals that some features might be indicative of the adversarial points.

#### 5.2.1 Multiple linear regression analysis.

To formally test this idea, multiple linear regression analysis is used, specifically the scikit-learn implementation. For each object in the training set, *N* points with the highest adversarial score *z*(**p**_*i*_) are selected, and then the regression model (13) is fitted.

Hypothesis test (14) is run for each coefficient *c*_*j*_ to see whether the null hypothesis can be rejected at the significance level of α=0.05. Those coefficients for which the null hypothesis can be rejected are deemed significantly different from zero, and the corresponding feature is regarded as sufficiently explanatory for *z*(**p**_*i*_). Other coefficients, for which the null hypothesis cannot be rejected, are deemed insignificant. This procedure is repeated for N∈{50,100,150,200}.

This linear analysis enables statistical interpretation of individual features and identifies those that are significantly associated with the adversarial score under a linear assumption.

The results are shown in Tables 2–4 for PointNet, PointNet++, and DGCNN, respectively, where insignificant coefficients are shown as 0.

**Table 2:** Multiple linear regression analyses for N∈{50,100,150,200} points with the highest adversarial score derived from PointNet. Significant coefficients (at α=0.05) are shown with 3 decimal points precision, while insignificant coefficients are shown as 0.

*N*	*c*_1_	*c*_2_	*c*_3_	*c*_4_	*c*_5_	*c*_6_	*c*_7_	*c*_8_	*c*_9_	*c*_10_	*c*_11_	*c*_12_	*c*_13_	*c*_14_	*R*^2^ (%)
50	−38.043	0.007	0.005	−0.009	0	0	0	0	4.659	0.648	0.011	−3.554	12.309	0.196	94.3
100	−44.032	0.007	0.006	−0.008	0	0	0	0	5.113	0.636	0.011	−3.139	11.733	0.164	94.2
150	−42.295	0.007	0.006	−0.007	0	0	0	0	4.904	0.623	0.010	−3.055	11.470	0.160	94.1
200	−41.451	0.007	0.005	−0.007	0	0	0	0	4.819	0.611	0.010	−2.969	11.207	0.153	93.9

**Table 3:** Multiple linear regression analyses for N∈{50,100,150,200} points with the highest adversarial score derived from PointNet++. Significant coefficients (at α=0.05) are shown with 3 decimal points precision, while insignificant coefficients are shown as 0.

*N*	*c*_1_	*c*_2_	*c*_3_	*c*_4_	*c*_5_	*c*_6_	*c*_7_	*c*_8_	*c*_9_	*c*_10_	*c*_11_	*c*_12_	*c*_13_	*c*_14_	*R*^2^ (%)
50	−54.854	0.009	0.006	−0.007	0	0	0	8.859	6.112	0.649	0.011	−2.665	11.375	0.122	94.3
100	−49.452	0.008	0.005	−0.007	0	0	0	6.851	5.544	0.636	0.010	−2.908	11.370	0.148	94.2
150	−44.927	0.008	−0.006	−0.008	0	0	0	3.120	5.125	0.624	0.010	−3.067	11.320	0.161	94.1
200	−43.109	0.007	0.005	−0.007	0	0	0	2.489	4.938	0.612	0.010	−3.057	11.091	0.163	93.9

**Table 4:** Multiple linear regression analyses for N∈{50,100,150,200} points with the highest adversarial score derived from DGCNN. Significant coefficients (at α=0.05) are shown with a precision of 3 decimal points, while insignificant coefficients are shown as 0.

*N*	*c*_1_	*c*_2_	*c*_3_	*c*_4_	*c*_5_	*c*_6_	*c*_7_	*c*_8_	*c*_9_	*c*_10_	*c*_11_	*c*_12_	*c*_13_	*c*_14_	*R*^2^ (%)
50	−52.555	0.006	0.006	−0.008	0	0	0	10.169	5.870	0.648	0.011	−3.058	11.745	0.157	94.4
100	−46.105	0.006	0.005	−0.007	0	0	0	4.473	5.241	0.636	0.011	−3.257	11.818	0.177	94.3
150	−43.374	0.007	0.005	−0.007	0	0	0	3.352	4.960	0.623	0.011	−3.179	11.540	0.173	94.2
200	−41.795	0.006	0.004	−0.007	0	0	0	3.585	4.806	0.610	0.011	−3.153	11.330	0.174	94.0

Consider Table 2 first. Four coefficients are insignificant: *c*_5_, *c*_6_, *c*_7_, and *c*_8_, and these are shown as 0, while the others are significant at α=0.05. The last column contains the *R*^2^ coefficient of determination [67], shown as a percentage. *R*^2^ is around 94%, which is fairly high and indicates good agreement between the data and the model

As the number of points *N* increases, it becomes harder for the model to fit the data, hence the drop in *R*^2^. Similar behavior can be seen in Tables 3 and 4 as well, although in these cases, *c*_8_ is significant.

Another observation is that there is a large overlap between the set of significant coefficients for PointNet, PointNet++, and DGCNN, even though these DNN models are quite different. This suggests that certain features of the point cloud itself may be able to predict adversarial points for different DNN models, and this is what makes new attacks possible. Specifically, we see that edge intensity (*f*_1_), weighted coordinate-based features (*f*_2_, *f*_3_, *f*_4_), local variation-based feature (*f*_9_), distance from the centroid (*f*_10_), and LPF-based features (*f*_11_, *f*_12_, *f*_13_, *f*_14_) are all indicative of the adversarial score for all three DNN models.

Rather than focusing on precise numerical prediction of the adversarial score, the multiple linear regression analysis is primarily used to identify which intrinsic geometric features consistently contribute to adversarial vulnerability. The results in Tables 2–4 reveal a strong overlap in the set of statistically significant features across different network architectures and different values of *N*.

This observation indicates that the main utility of the linear regression model lies in robust feature identification and relative ranking of vulnerable points, rather than exact score estimation. Such a ranking is sufficient for constructing effective drop-based attacks, since only the ordering of points according to their vulnerability is required.

Although the exact coefficient values vary across different values of *N* and different architectures, the set of significant features remains largely stable, suggesting that these features capture intrinsic geometric properties related to adversarial vulnerability.

The consistency of these findings motivates further validation using a non-linear regression model, which is discussed in the following subsection.

#### 5.2.2 Random forest regression analysis.

**Fig 4:**
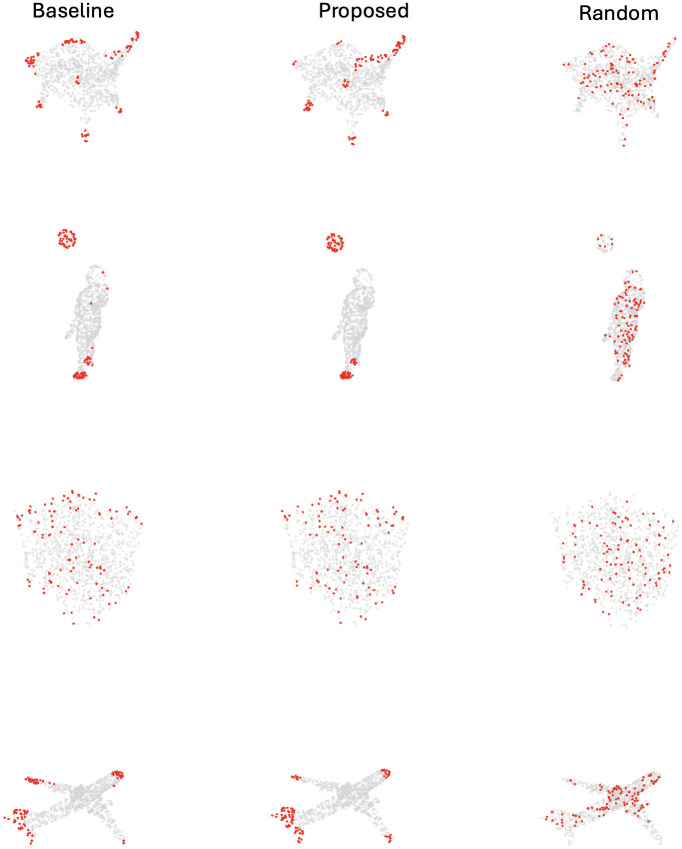
Feature importance obtained from random forest regression for N∈{50,100,150,200} points with the highest adversarial score derived from PointNet, PointNet++, and DGCNN. The distance from centroid feature (*f*_10_) consistently exhibits the highest importance across all architectures, indicating its dominant role under non-linear modeling.

Unlike multiple linear regression, random forest regression allows for non-linear interactions between features and does not rely on explicit model assumptions, making it suitable as a validation tool. The analysis is performed using the same adversarial scores and geometric features defined in this work. For each point cloud in the training set, a random forest regressor is trained to predict the adversarial score *z*(**p**_*i*_) from the fourteen geometric features. Instead of regression coefficients, the random forest model provides feature importance scores, which quantify the relative contribution of each feature to the prediction based on the average reduction in prediction error across the ensemble of decision trees. Fig 4 shows the resulting feature importance distributions obtained from random forest regression for N∈{50,100,150,200}, reported separately for adversarial scores computed from PointNet, PointNet++, and DGCNN. Each bar represents the normalized importance of a feature under non-linear modeling. A clear and consistent trend can be observed across all three network architectures and all values of *N*. In particular, the distance-from-centroid feature (*f*_10_) consistently receives the highest importance score, indicating its dominant role in predicting adversarially vulnerable points. Other features exhibit substantially lower and less stable importance values. The strong agreement between the non-linear feature importance rankings and the linear analysis supports the conclusion that adversarial vulnerability is primarily governed by intrinsic geometric properties of the point cloud. This observation motivates the use of the distance-from-centroid feature as the core cue for constructing the feature-driven drop attack described in the next subsection.

### 5.3 Attack success rate

The attack success rate measures how effective a particular adversarial attack is at fooling the target deep model. The attack success rate is typically reported as a percentage of cases in which the target model was fooled on a given dataset, with a higher number indicating a more successful attack. The next experiment examines the attack success rates on the ModelNet40 test set.

Since there are no existing attacks based on intrinsic characteristics of the point clouds in 3D point cloud classification, we pick as our ”Baseline” method an attack based on saliency score (1), which is the un-normalized version of the true adversarial score (12). The ”Baseline” attack involves dropping 100 or 200 points with the highest true saliency scores. This is a white-box attack and requires access to the target DNN model so that gradients relative to the loss function can be computed in (1). Although the white-box attack clearly has an advantage over the proposed attack, the comparison still helps us gain insight into how much the attack success rate can be improved by having direct access to the target DNN model.

The second method (“Proposed”) is the proposed attack, which drops 100 or 200 points with the highest predicted adversarial score $𝔷({\text{p}}_{i})$ from (16).

The third method (“Random”) drops 100 or 200 randomly selected points.

Table 5 shows the attack success rate results. As expected, the Baseline method achieves the highest success rate in most cases, since it is a white-box attack that uses a particular target DNN model to identify the most influential points for that model.

**Table 5:** Comparison of the Baseline, Proposed, and Random attacks in terms of attack success rate.

Model	Attacks					
	Baseline		Proposed		Random	
	Drop100 [24]	Drop200 [24]	Drop100	Drop200	Random Drop100	Random Drop200
PointNet [33]	**33.00**	**49.84**	22.70	28.81	19.00	18.20
PointNet++ [34]	**16.50**	**25.57**	16.01	21.84	11.83	11.51
DGCNN [69]	**18.19**	**29.98**	13.98	18.76	**10.86**	11.02
PointConv [36]	**10.86**	12.24	10.09	**12.40**	8.14	8.39

However, the Proposed attack, exhibits performance that is generally comparable to the Baseline on PointNet++, DGCNN, and PointConv.

Moreover, its performance across different DNN models is fairly consistent; except for the PointNet architecture, where a larger performance gap between the Proposed and Baseline methods is observed. For PointNet++, DGCNN, and PointConv, both methods demonstrate similar trends across Drop100 and Drop200 settings. Notably, in the Drop200 setting on PointConv, the Proposed attack slightly outperforms the Baseline. Overall, these results indicate that an attack constructed solely from intrinsic geometric cues can achieve performance comparable to architecture-dependent white-box methods across a range of network models.

### 5.4 Transferability

When adversarial points are determined for a particular DNN model, it is natural to ask whether the same adversarial points could create a successful attack on another DNN model. The ability to transfer an attack from one DNN model to another is an important aspect to consider when evaluating the robustness of an attack strategy. In this experiment, we examine how well the adversarial points for the Drop100 and drop200 attacks identified on one DNN model (”source”) work on another DNN model (”target”).

Tables 6 and 7 show the transferability results for PointConv in terms of the success rate of the Drop100 and Drop200 attacks on the ModelNet40 test set. When the source and target models match, the Baseline approach has a higher success rate than the Proposed method, as expected.

**Table 6:** Transferability of Drop100 attacks in terms of attack success rate.

		Target DNN model			
Source DNN Model	Drop100 Attack	PointNet [33]	PointNet++ [34]	DGCNN [69]	PointConv [36]
PointNet [33]	Baseline [24]	**33.00**	19.09	19.41	18.31
	Proposed (coefficients)	22.70	**22.70**	**22.70**	**22.70**
PointNet++ [34]	Baseline [24]	14.43	**16.50**	13.09	12.07
	Proposed (coefficients)	**16.01**	16.01	**16.01**	**16.01**
DGCNN [69]	Baseline [24]	**13.70**	**13.29**	**18.19**	12.40
	Proposed (coefficients)	12.97	12.97	12.97	**12.97**
PointConv [36]	Baseline [24]	9.64	8.83	9.44	**10.86**
	Proposed (coefficients)	**10.09**	**10.09**	**10.09**	10.09

**Table 7:** Transferability of Drop200 attacks in terms of attack success rate.

		Target DNN model			
Source DNN Model	Drop200 Attack	PointNet [33]	PointNet++ [34]	DGCNN [69]	PointConv [36]
PointNet [33]	Baseline [24]	**49.84**	20.87	20.83	18.52
	Proposed (coefficients)	28.81	**28.81**	**28.819**	**28.81**
PointNet++ [34]	Baseline [24]	19.21	**25.69**	16.78	12.48
	Proposed (coefficients)	**20.95**	20.95	**20.95**	**20.95**
DGCNN [69]	Baseline [24]	**19.37**	**19.89**	**29.98**	13.49
	Proposed (coefficients)	18.76	18.76	18.76	**18.76**
PointConv [36]	Baseline [24]	11.06	10.78	11.43	**12.24**
	Proposed (coefficients)	**12.40**	**12.40**	**12.40**	12.40

Since the Proposed attack is constructed independently of the source model and relies only on the intrinsic geometric cue *f*_10_, its attack success rate remains unchanged across different target architectures for a fixed Drop-*N* setting. In contrast, the Baseline approach, which relies on model-specific saliency information, generally degrades when transferred to a different target model, and its performance can vary substantially across mismatched source–target pairs. Overall, the Proposed approach exhibits higher transferability and a more predictable performance against an unknown target model, achieving on average around a 2% higher success rate for Drop100 and a 4% higher success rate for Drop200 relative to the Baseline in transferability experiments.

### 5.5 Performance of various defenses

Next, we test the performance of several defenses – SRS [27], SOR [30], DUP-Net [30], and [28] LPF-Defense [29]– against the Baseline and Proposed Drop100 and drop200 attacks. Table 8 shows the classification accuracy of PointConv under different attacks and defenses. For reference, the ”No-attack” column shows the accuracy when there is no attack. The lower classification accuracy indicates that the attack is more successful.

**Table 8:** Performance of various defenses against Drop100 and Drop200 attacks in terms of PointConv classification accuracy; lower classification accuracy indicates a more successful attack.

		Drop100 Attack		Drop200 Attack	
Defense	No-attack	Baseline	Proposed	Baseline	Proposed
No-defense	91.82%	89.14%	89.91%	81.81%	86.02%
SRS [27]	91.33%	89.02%	**88.57%**	87.56%	**85.41%**
SOR [30]	91.57%	88.78%	**88.74%**	87.88%	**84.76%**
DUP-Net [30]	85.37%	84.68%	**82.90%**	84.16%	**80.88%**
LPF-Defense [29]	90.32%	**87.97%**	88.45%	86.35%	**84.48%**

As shown in Table 8, the Proposed attack leads to lower classification accuracy than the Baseline attack across most of the evaluated defenses, for both Drop100 and Drop200 attacks. This observation suggests that the geometric criterion employed by the Proposed method is not fully aligned with the underlying assumptions of common defense mechanisms, thereby partially limiting their effectiveness. In contrast, the Baseline attack relies on model-dependent saliency information, which can be partially neutralized by defense strategies specifically designed to mitigate gradient-based perturbations.

### 5.6 Computational cost

Next, the computational cost of generating the Baseline and Proposed Drop100 attacks is compared. Again, the Proposed attack is based on the the distance-from-centroid feature (*f*_10_), which was consistently identified as the most informative intrinsic geometric feature across both linear and non-linear analyses. The experiment was carried out in a Google Colab environment using an NVIDIA Tesla T4 GPU. Table 8 shows the average run time in seconds per point cloud needed to generate a Drop100 attack, averaged over the ModelNet40 test set. As seen in the Table 9, the Proposed attack is approximately 331 times faster than the Baseline.

**Table 9:** Average run-time for generating an adversarial point cloud in a Drop100 attack.

Drop100 Attack	Time (s)
Baseline	0.036
Proposed	0.00011

**Computational Efficiency.** The proposed method consists of multiple stages—including handcrafted graph-based feature extraction, score computation, and point removal—but all steps are computationally lightweight and involve no iterative optimization or gradient computation during inference. Unlike gradient-based methods, which require repeated forward and backward passes through the model for each input, the proposed approach relies on precomputed geometric features and a simple ranking-based selection procedure. The feature extraction step is performed once per input point cloud and is used as input to a lightweight scoring and selection process, which avoids repeated per-model computations. This results in high efficiency in both runtime and memory usage. Despite these simplifications, the attack remains consistently effective across both lightweight and heavyweight classifiers.

### 5.7 Visualizations

**Fig 5:**
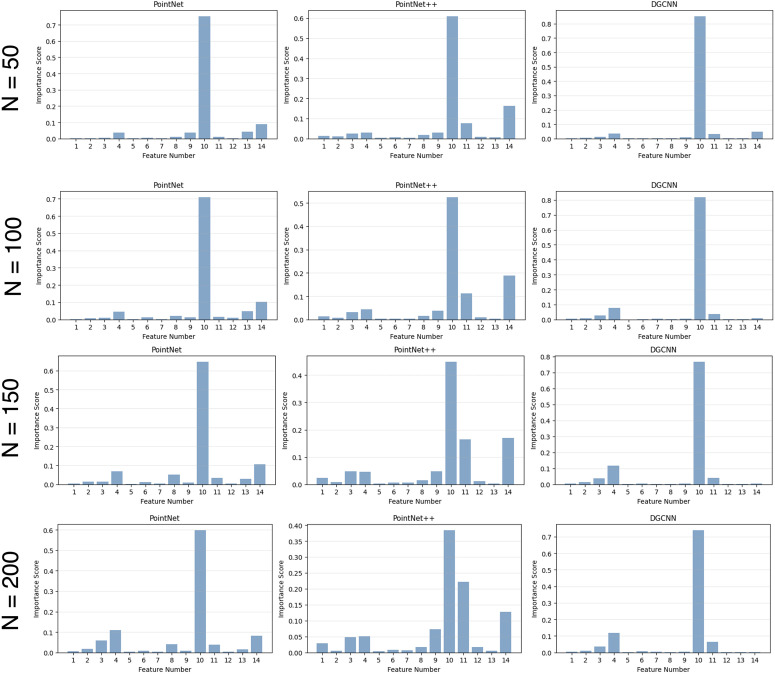
Visualisation of adversarial point prediction, where (predicted) adversarial points are shown in red, and the other points in the cloud as gray. (Left) 100 points with the highest true adversarial score computed based on PointNet. (Middle) 100 points with the highest predicted adversarial score computed using the proposed geometric feature *f*_10_. (Right) 100 randomly selected points.

Several visualizations of *true and predicted adversarial drop points* are presented in Fig 5. The left column displays the 100 points with the highest adversarial score *z*(**p**_*i*_) from (12) computed using PointNet, representing the *true adversarial drop points*, shown in red; all remaining points are rendered in gray. The middle column illustrates the 100 *predicted adversarial drop points* with the highest score $𝔷({\text{p}}_{i})$ according to (16), The right column highlights 100 randomly selected points.

A qualitative comparison between the left and middle columns reveals a strong agreement between the true and predicted adversarial drop points. In all examples—including the chair (top row), human (second row), Xbox (third row), and airplane (bottom row) —both sets of points are concentrated in geometrically salient regions such as edges, corners, and structural joints. Notably, the predicted points in the middle column not only closely resemble the true adversarial drop points but also exhibit a stronger preservation of global symmetry. For instance, in the airplane example, the predicted drops are distributed symmetrically across both wings, while the true drops (based on the white-box saliency method) concentrate more heavily on a single wing, causing visible asymmetry. This enhanced structural plausibility of the predicted adversarial drop points improves stealthiness in real-world scenarios by avoiding the unnatural asymmetries often introduced by white-box methods, thereby making adversarial manipulations harder to detect.

## 6 Conclusion

In this paper, we investigated the role of intrinsic point cloud features in crafting 3D adversarial attacks, shifting the focus from model-specific vulnerabilities to the intrinsic characteristics of point clouds. By defining a set of fourteen features derived from graph signal processing concepts, features that significantly influence adversarial drop points—points whose removal is likely to alter a model’s decision—were identified.

Through combined multiple linear regression and random forest analyses, we identified the distance from the centroid as a consistently dominant geometric feature associated with adversarial vulnerability across multiple DNN models, including PointNet, PointNet++, DGCNN, and PointConv.

The findings indicate that adversarial vulnerability in point clouds is not a flaw of deep network architectures but is also inherently linked to the geometric structure of the data itself. Based on these insights, a novel attack method was developed. While this new attack does not surpass white-box methods that are explicitly optimized for a specific target model, it demonstrates strong generalization across architectures, higher transferability, and significantly lower computational cost compared to a baseline white-box attack.

Beyond adversarial attack development, this study introduces a novel perspective in deep learning that shifts the focus from gradient-based optimization to decision-making based on intrinsic data features. This shift not only provides new insights into how deep neural networks process point cloud data but also paves the way for more interpretable, faster, and computationally efficient learning models. Moreover, the proposed approach exemplifies a combination novelty by unifying concepts from 3D geometry, adversarial learning, and graph signal processing in a model-independent and interpretable setting.

Our approach establishes a basis for future research in enhancing adversarial robustness and improving model explainability, ultimately contributing to the design of more effective and transferable attack and defense mechanisms in point cloud-based applications such as robotics, autonomous driving, and 3D object recognition.

## 7 Future direction

Given the lightweight and interpretable nature of the proposed feature-based scoring function, future work could explore its potential for defense applications. One possible direction is to build a statistical profile of clean point clouds based on their feature scores. At inference time, if a new input contains an unusually high number of points with abnormal scores, it could be flagged as adversarial or unreliable. This lightweight preprocessing step could improve robustness without modifying the model architecture. Another promising direction is extending this framework to dynamic 4D spatiotemporal point clouds (e.g., LiDAR sequences), enabling adversarial analysis in real-world, time-dependent environments.

## References

[1] Zhang K, Zuo W, Chen Y, Meng D, Zhang L. Beyond a Gaussian Denoiser: Residual Learning of Deep CNN for Image Denoising. IEEE Trans Image Process. 2017;26(7):3142–55. doi: 10.1109/TIP.2017.2662206 28166495

[2] Song Y, He Z, Qian H, Du X. Vision Transformers for Single Image Dehazing. IEEE Trans Image Process. 2023;32:1927–41. doi: 10.1109/TIP.2023.3256763 37030760

[3] Kyong Hwan Jin, McCann MT, Froustey E, Unser M. Deep Convolutional Neural Network for Inverse Problems in Imaging. IEEE Trans Image Process. 2017;26(9):4509–22. doi: 10.1109/TIP.2017.2713099 28641250

[4] Dong Y, Liu Q, Du B, Zhang L. Weighted Feature Fusion of Convolutional Neural Network and Graph Attention Network for Hyperspectral Image Classification. IEEE Trans Image Process. 2022;31:1559–72. doi: 10.1109/TIP.2022.3144017 35077363

[5] Naderi H, Goli L, Kasaei S. Scale Equivariant CNNs with Scale Steerable Filters. In: International Conference on Machine Vision and Image Processing (MVIP). 2020. p. 1–5.

[6] Goodfellow IJ, Shlens J, Szegedy C. Explaining and harnessing adversarial examples. In: International Conference on Learning Representations (ICLR). 2015.

[7] Szegedy C, Zaremba W, Sutskever I, Bruna J, Erhan D, Goodfellow IJ, et al. Intriguing Properties of Neural Networks. In: International Conference on Learning Representations (ICLR). 2014.

[8] Moosavi-Dezfooli SM, Fawzi A, Fawzi O, Frossard P. Universal Adversarial Perturbations. In: Proceedings of the IEEE Conference on Computer Vision and Pattern Recognition (CVPR). 2017. p. 1765–73.

[9] Carlini N, Wagner D. Towards Evaluating the Robustness of Neural Networks. In: 2017 IEEE Symposium on Security and Privacy (SP). 2017. p. 39–57.

[10] Naderi H, Goli L, Kasaei S. Generating unrestricted adversarial examples via three parameteres. Multimed Tools Appl. 2022;81(15):21919–38. doi: 10.1007/s11042-022-12007-x

[11] Liu D, Yu R, Su H. Extending Adversarial Attacks and Defenses to Deep 3D Point Cloud Classifiers. In: 2019 IEEE International Conference on Image Processing (ICIP). 2019. p. 2279–83.

[12] Xiang C, Qi CR, Li B. Generating 3D Adversarial Point Clouds. In: 2019 IEEE/CVF Conference on Computer Vision and Pattern Recognition (CVPR). 2019. p. 9128–36.

[13] Huang Q, Dong X, Chen D, Zhou H, Zhang W, Yu N. Shape-invariant 3D Adversarial Point Clouds. In: 2022 IEEE/CVF Conference on Computer Vision and Pattern Recognition (CVPR), 2022. p. 15314–23.

[14] Wen C, Li X, Huang H, Liu Y-S, Fang Y. 3D Shape Contrastive Representation Learning With Adversarial Examples. IEEE Trans Multimedia. 2025;27:679–92. doi: 10.1109/tmm.2023.3265177

[15] Naderi H, Bajić IV. Adversarial Attacks and Defenses on 3D Point Cloud Classification: A Survey. IEEE Access. 2023;11:144274–95. doi: 10.1109/access.2023.3345000

[16] Liu D, Hu W, Li X. Point Cloud Attacks in Graph Spectral Domain: When 3D Geometry Meets Graph Signal Processing. IEEE Trans Pattern Anal Mach Intell. 2024;46(5):3079–95. doi: 10.1109/TPAMI.2023.3339130 38051619

[17] Huang Q, Dong X, Chen D, Zhou H, Zhang W, Zhang K, et al. PointCAT: Contrastive Adversarial Training for Robust Point Cloud Recognition. IEEE Trans Image Process. 2024;33:2183–96. doi: 10.1109/TIP.2024.3372456 38451765

[18] Zhang K, Zhou H, Zhang J, Huang Q, Zhang W, Yu N. Ada3Diff: Defending against 3D Adversarial Point Clouds via Adaptive Diffusion. In: Proceedings of the 31st ACM International Conference on Multimedia. 2023. p. 8849–59.

[19] Hamdi A, Rojas S, Thabet A, Ghanem B. AdvPC: Transferable Adversarial Perturbations on 3D Point Clouds. In: Lecture Notes in Computer Science. Springer International Publishing; 2020. p. 241–57. 10.1007/978-3-030-58610-2_15

[20] Cheng S, Chen X, He X, Liu Z, Bai X. PRA-Net: Point Relation-Aware Network for 3D Point Cloud Analysis. IEEE Trans Image Process. 2021;30:4436–48. doi: 10.1109/TIP.2021.3072214 33856993

[21] Lu T, Liu C, Chen Y, Wu G, Wang L. APP-Net: Auxiliary-Point-Based Push and Pull Operations for Efficient Point Cloud Recognition. IEEE Trans Image Process. 2023;32:6500–13. doi: 10.1109/TIP.2023.3333191 37988214

[22] de Queiroz RL, Chou PA. Compression of 3D Point Clouds Using a Region-Adaptive Hierarchical Transform. IEEE Trans Image Process. 2016;25(8):3947–56. doi: 10.1109/TIP.2016.2575005 27254868

[23] Dinesh C, Cheung G, Bajic IV. Point Cloud Denoising via Feature Graph Laplacian Regularization. IEEE Trans Image Process. 2020;29:4143–58. doi: 10.1109/TIP.2020.2969052 32012012

[24] Zheng T, Chen C, Yuan J, Li B, Ren K. PointCloud Saliency Maps. In: Proceedings of the IEEE/CVF International Conference on Computer Vision (ICCV). 2019. p. 1598–606.

[25] Zhang B, Huang S, Shen W, Wei Z. Explaining the PointNet: What has been learned inside the PointNet? In: Proceedings of the IEEE/CVF Conference on Computer Vision and Pattern Recognition Workshops. 2019. p. 71–4.

[26] Fan S, Gao W, Li G. Salient Object Detection for Point Clouds. arXiv preprint arXiv:220711889. 2022.

[27] Yang J, Zhang Q, Fang R, Ni B, Liu J, Tian Q. Adversarial Attack and Defense on Point Sets. arXiv preprint arXiv:190210899. 2021.

[28] Wu Z, Duan Y, Wang H, Fan Q, Guibas LJ. IF-Defense: 3D Adversarial Point Cloud Defense via Implicit Function Based Restoration. arXiv preprint arXiv:201005272. 2020.

[29] Naderi H, Noorbakhsh K, Etemadi A, Kasaei S. LPF-Defense: 3D adversarial defense based on frequency analysis. PLoS One. 2023;18(2):e0271388. doi: 10.1371/journal.pone.0271388 36745627 PMC9901796

[30] Zhou H, Chen K, Zhang W, Fang H, Zhou W, Yu N. DUP-Net: Denoiser and Upsampler Network for 3D Adversarial Point Clouds Defense. In: 2019 IEEE/CVF International Conference on Computer Vision (ICCV). 2019. p. 1961–70.

[31] Jamali N, Sani MM, Naderi H, Kasaei S. KNN-Defense: Defense Against 3D Adversarial Point Clouds Using Nearest-Neighbor Search. arXiv preprint arXiv:250606906. 2025.

[32] Ilyas A, Santurkar S, Tsipras D, Engstrom L, Tran B, Madry A. Adversarial Examples Are Not Bugs, They Are Features. In: Advances in Neural Information Processing Systems (NeurIPS). 2019. p. 125–36.

[33] Charles RQ, Su H, Kaichun M, Guibas LJ. PointNet: Deep Learning on Point Sets for 3D Classification and Segmentation. In: 2017 IEEE Conference on Computer Vision and Pattern Recognition (CVPR). 2017. p. 77–85.

[34] Qi CR, Yi L, Su H, Guibas LJ. PointNet++: Deep Hierarchical Feature Learning on Point Sets in a Metric Space. arXiv preprint arXiv:170602413. 2017.

[35] Wang Y, Sun Y, Liu Z, Sarma SE, Bronstein MM, Solomon JM. Dynamic Graph CNN for Learning on Point Clouds. ACM Trans Graph. 2019;38(5):1–12. doi: 10.1145/3326362

[36] Wu W, Qi Z, Fuxin L. PointConv: Deep Convolutional Networks on 3D Point Clouds. In: 2019 IEEE/CVF Conference on Computer Vision and Pattern Recognition (CVPR). 2019. p. 9613–22.

[37] Fan L, He F, Si T, Tang W, Li B. Invisible Backdoor Attack against 3D Point Cloud Classifier in Graph Spectral Domain. AAAI. 2024;38(19):21072–80. doi: 10.1609/aaai.v38i19.30099

[38] Wang X, Li M, Xu P, Liu W, Zhang LY, Hu S. PointAPA: Towards Availability Poisoning Attacks in 3D Point Clouds. In: European Symposium on Research in Computer Security (ESORICS). Springer; 2024. p. 125–45.

[39] Madry A, Makelov A, Schmidt L, Tsipras D, Vladu A. Towards Deep Learning Models Resistant to Adversarial Attacks. arXiv preprint arXiv:170606083. 2017.

[40] Tsai T, Yang KH, Ho TY, Jin Y. Robust Adversarial Objects Against Deep Learning Models. In: Proceedings of the AAAI Conference on Artificial Intelligence. 2020. p. 954–62.

[41] Kim J, Hua B-S, Nguyen DT, Yeung S-K. Minimal Adversarial Examples for Deep Learning on 3D Point Clouds. In: 2021 IEEE/CVF International Conference on Computer Vision (ICCV). 2021. p. 7777–86.

[42] Wicker M, Kwiatkowska M. Robustness of 3D Deep Learning in an Adversarial Setting. In: 2019 IEEE/CVF Conference on Computer Vision and Pattern Recognition (CVPR). 2019. p. 11759–67.

[43] Arya A, Naderi H, Kasaei S. Adversarial Attack by Limited Point Cloud Surface Modifications. In: 2023 6th International Conference on Pattern Recognition and Image Analysis (IPRIA). 2023. p. 1–8.

[44] Fan L, He F, Song Y, Xu H, Li B. Look inside 3D point cloud deep neural network by patch-wise saliency map. ICA. 2024;31(2):197–212. doi: 10.3233/ica-230725

[45] Liu D, Yu R, Su H. Adversarial shape perturbations on 3D point clouds. In: European Conference on Computer Vision Workshops (ECCVW). 2020. p. 88–104.

[46] Zhao B, Hua X, Yu K, Tao W, He X, Feng S, et al. Evaluation of Convolution Operation Based on the Interpretation of Deep Learning on 3-D Point Cloud. IEEE J Sel Top Appl Earth Observations Remote Sensing. 2020;13:5088–101. doi: 10.1109/jstars.2020.3020321

[47] Tayyub J, Sarmad M, Schönborn N. Explaining Deep Neural Networks for Point Clouds Using Gradient-Based Visualisations. In: Proceedings of the Asian Conference on Computer Vision (ACCV). 2022. p. 2123–38.

[48] Arnold NI, Angelov P, Atkinson PM. An Improved Explainable Point Cloud Classifier (XPCC). IEEE Trans Artif Intell. 2023;4(1):71–80. doi: 10.1109/tai.2022.3150647

[49] Taghanaki SA, Hassani K, Jayaraman PK, Khasahmadi AH, Custis T. PointMask: Towards Interpretable and Bias-Resilient Point Cloud Processing. arXiv preprint arXiv:200704525. 2020.

[50] Zhang M, You H, Kadam P, Liu S, Kuo C-CJ. PointHop: An Explainable Machine Learning Method for Point Cloud Classification. IEEE Trans Multimedia. 2020;22(7):1744–55. doi: 10.1109/tmm.2019.2963592

[51] Shuman DI, Narang SK, Frossard P, Ortega A, Vandergheynst P. The emerging field of signal processing on graphs: Extending high-dimensional data analysis to networks and other irregular domains. IEEE Signal Process Mag. 2013;30(3):83–98. doi: 10.1109/msp.2012.2235192

[52] Liu X, Cheung G, Wu X, Zhao D. Random Walk Graph Laplacian-Based Smoothness Prior for Soft Decoding of JPEG Images. IEEE Trans Image Process. 2017;26(2):509–24. doi: 10.1109/TIP.2016.2627807 27849534

[53] Ortega A, Frossard P, Kovačević J, Moura JMF, Vandergheynst P. Graph Signal Processing: Overview, Challenges, and Applications. Proc IEEE. 2018;106(5):808–28. doi: 10.1109/jproc.2018.2820126

[54] Chen S, Tian D, Feng C, Vetro A, Kovacevic J. Fast Resampling of Three-Dimensional Point Clouds via Graphs. IEEE Trans Signal Process. 2018;66(3):666–81. doi: 10.1109/tsp.2017.2771730

[55] Cheung G, Magli E, Tanaka Y, Ng MK. Graph Spectral Image Processing. Proc IEEE. 2018;106(5):907–30. doi: 10.1109/jproc.2018.2799702

[56] Dinesh C, Cheung G, Bajic IV. Super-Resolution of 3D Color Point Clouds Via Fast Graph Total Variation. In: ICASSP 2020 - 2020 IEEE International Conference on Acoustics, Speech and Signal Processing (ICASSP). 2020. p. 1983–7.

[57] Dinesh C, Cheung G, Bajić IV. 3D Point Cloud Color Denoising Using Convex Graph-Signal Smoothness Priors. In: 2019 IEEE 21st International Workshop on Multimedia Signal Processing (MMSP). 2019. p. 1–6.

[58] Dinesh C, Cheung G, Wang F, Bajic IV. Sampling of 3D Point Cloud via Gershgorin Disc Alignment. In: Proceedings of the IEEE International Conference on Image Processing (ICIP). 2020. p. 2736–40.

[59] Hu W, Gao X, Cheung G, Guo Z. Feature Graph Learning for 3D Point Cloud Denoising. IEEE Trans Signal Process. 2020;68:2841–56. doi: 10.1109/tsp.2020.2978617 32012012

[60] Fu Z, Hu W, Guo Z. 3D Dynamic Point Cloud Inpainting via Temporal Consistency on Graphs. In: Proceedings of the IEEE International Conference on Multimedia and Expo (ICME). 2020. p. 1–6.

[61] Qi J, Hu W, Guo Z. Feature Preserving and Uniformity-Controllable Point Cloud Simplification on Graph. In: Proceedings of the IEEE International Conference on Multimedia and Expo (ICME). 2019. p. 284–9.

[62] Zeng J, Cheung G, Ng MK, Pang J, Yang C. 3D Point Cloud Denoising Using Graph Laplacian Regularization of a Low Dimensional Manifold Model. IEEE Transactions on Image Processing. 2020;29:3474–89. 10.1109/TIP.2019.2961429 31899426

[63] Dinesh C, Cheung G, Bajic IV. Point Cloud Sampling via Graph Balancing and Gershgorin Disc Alignment. IEEE Trans Pattern Anal Mach Intell. 2023;45(1):868–86. doi: 10.1109/TPAMI.2022.3143089 35025739

[64] Dinesh C, Cheung G, Bajic IV. Point Cloud Video Super-Resolution via Partial Point Coupling and Graph Smoothness. IEEE Trans Image Process. 2022;31:4117–32. doi: 10.1109/TIP.2022.3166644 35696478

[65] Schoenenberger Y, Paratte J, Vandergheynst P. Graph-Based Denoising for Time-Varying Point Clouds. In: IEEE 3DTV-Conference; 2015. p. 1–4.

[66] Chung FRK, Graham FC. Spectral Graph Theory. vol. 92. American Mathematical Society; 1997.

[67] Kutner MH, Nachtsheim CJ, Neter J, Li W. Applied Linear Statistical Models. 5th edition. McGraw-Hill/Irwin; 2005.

[68] Eberly LE. Multiple linear regression. Topics in biostatistics. 2007. p. 165–87. 10.1007/978-1-59745-530-5_9 18450050

[69] Phan AV, Nguyen ML, Nguyen YLH, Bui LT. DGCNN: A convolutional neural network over large-scale labeled graphs. Neural Netw. 2018;108:533–43. doi: 10.1016/j.neunet.2018.09.001 30458952

